# Magnetocardiographic characteristics in patients with varying severity of obstructive sleep apnea syndrome

**DOI:** 10.3389/fmed.2026.1932236

**Published:** 2026-10-15

**Authors:** Liang Liu, Zhen-Fa Zhou, Yu-Jie Zhang, Yu-Pu Liu, Yue-Li Zhang, Jian Ma, Cheng-Xing Shen

**Affiliations:** 1 Department of Cardiology, Shanghai Sixth People’s Hospital Affiliated to Shanghai Jiao Tong University School of Medicine, Shanghai, China; 2 Department of Cardiology, Fuzhou First General Hospital Affiliated with Fujian Medical University, Fuzhou, China; 3 Department of Otorhinolaryngology–Head and Neck Surgery, Shanghai Sixth People’s Hospital Affiliated to Shanghai Jiao Tong University School of Medicine, Shanghai, China; 4 Shanghai Key Laboratory of Sleep Disordered Breathing, Shanghai Sixth People’s Hospital Affiliated to Shanghai Jiao Tong University School of Medicine, Shanghai, China; 5 Department of Ultrasound in Medicine, Shanghai Sixth People’s Hospital Affiliated to Shanghai Jiao Tong University School of Medicine, Shanghai, China

**Keywords:** magnetocardiography (MCG), obstructive sleep apnea syndrome (OSAS), cardiac diastolic function, coronary microcirculation disorder (CMD), predictive parameters

## Abstract

**Background:**

Obstructive sleep apnea syndrome (OSAS) is an established risk factor for cardiovascular disease. Magnetocardiography (MCG) is an emerging non-invasive electrophysiological technique with potential value in cardiovascular assessment; however, its utility in evaluating OSAS severity remains unclear. This study aimed to characterize MCG features across different severities of OSAS and to evaluate their predictive performance for moderate-to-severe disease.

**Methods:**

This retrospective study enrolled OSAS patients between June 2023 and October 2024, all of whom underwent MCG examination. Patients were stratified by OSAS severity, and MCG parameters were compared between the normal-to-mild and moderate-to-severe groups. Baseline data, complete blood count, serum biochemical indicators, and MCG parameters were collected along with relevant clinical and laboratory data. Univariate and multivariate logistic regression analyses were performed to identify independent predictors of moderate-to-severe OSAS, and receiver operating characteristic (ROC) curves were constructed to evaluate the predictive performance of each parameter and the combined model.

**Results:**

Group comparisons revealed significant differences in traditional OSAS severity risk factors (sex, body mass index, etc.). Diastolic function indicators, including E/A ratio and left atrial size, also showed marked differences between groups, with the moderate-to-severe subgroup exhibiting larger left atria and a higher proportion of E/A ratio values below 1. Univariate analysis identified 35 MCG parameters that differed between groups, while multivariate regression retained four independent predictors (htrt, tmdf, RS-didma, and qr_rela_dist_neg_mode). ROC analysis indicated that T-wave- and QRS-wave-related indices had moderate predictive value; a combined model incorporating htrt, tmdf, and RS-didma showed superior area under the curve compared to any single parameter.

**Conclusion:**

MCG parameters demonstrate distinct characteristics across OSAS severity levels and yield favorable predictive performance for moderate-to-severe OSAS, positioning MCG as a promising non-invasive tool for early diagnosis. The observed changes in diastolic function markers may suggest underlying coronary microcirculatory alterations in patients with moderate-to-severe OSAS. Rigorously designed prospective studies are warranted to validate MCG as a standardized diagnostic modality for OSAS-related coronary microcirculation disorder.

## 1 Introduction

Obstructive sleep apnea syndrome (OSAS) is a highly prevalent sleep disorder characterized by recurrent upper airway collapse during sleep, leading to intermittent hypoxia (IH), sleep fragmentation, and autonomic dysfunction (1). According to the diagnostic criteria established by the American Academy of Sleep Medicine (AASM), the global burden of OSAS is substantial, with an estimated 936 million individuals aged 30–69 years affected worldwide (2). However, owing to variations in race, sex, body habitus, and regional diagnostic capacity, the true prevalence is likely underestimated (3). OSAS is an independent risk factor for cardiovascular diseases, including atrial fibrillation and heart failure, and contributes significantly to cardiovascular mortality (4). Consequently, developing effective strategies to prevent and manage cardiovascular complications in OSAS patients has become an urgent public health priority.

Cardiovascular disease remains the leading cause of death globally, with ischemic cardiomyopathy representing a major clinical subtype primarily driven by underlying coronary artery disease (CAD) (5). Coronary microvascular dysfunction (CMD) also plays a critical role in impairing myocardial perfusion and exacerbating ischemic injury (6). Accumulating evidence confirms that OSAS is an independent risk factor for the development and progression of CAD (7). Moreover, OSAS is strongly associated with non-obstructive CMD, particularly in patients with moderate-to-severe OSAS. These patients frequently exhibit coronary endothelial dysfunction, resulting in microcirculatory impairment and subsequent myocardial hypoperfusion—a key pathophysiological mechanism in ischemic cardiomyopathy (8, 9). Therefore, timely evaluation of coronary microvascular function in moderate-to-severe OSAS patients is essential to guide early interventions that may delay the progression of ischemic cardiomyopathy.

Despite the established link between OSAS and CMD, current non-invasive diagnostic approaches for detecting CMD remain suboptimal, while invasive methods are limited by procedural and patient burden (10). In routine clinical practice, there remains a pressing unmet need for non-invasive, easy-to-administer, and sufficiently accurate tools capable of early identification of patients with moderate-to-severe OSAS, while also identifying actionable predictive biomarkers for subclinical CMD in this high-risk population. Magnetocardiography (MCG), a non-contact imaging technique that directly records the magnetic fields generated by intrinsic cardiac electrical activity, has emerged as a highly promising functional cardiac assessment modality, with particular diagnostic strengths in evaluating CMD-related myocardial perfusion abnormalities. Recent studies indicate that MCG may surpass ECG in detecting ischemic changes, offering the advantages of being radiation-free and highly sensitive to subtle electrophysiological alterations (11–13). To date, however, no published systematic investigations have characterized the full distribution of distinct magnetocardiographic signatures across clinically defined OSAS severity strata.

Against this clinical backdrop, the present study was designed to characterize MCG features across different OSAS severities and to evaluate their predictive performance for moderate-to-severe disease. We also describe the distribution of echocardiographic diastolic markers across OSAS severity subgroups as ancillary observations. By characterizing OSAS-specific MCG phenotypic patterns in this cohort, we aim to establish a convenient, non-invasive approach to support early risk stratification in OSAS patients at high cardiovascular risk. The findings of our study also provide a critical methodological and preliminary data foundation for subsequent large-scale investigations aimed at clarifying specific MCG-derived biomarkers for occult CMD in the OSAS population.

## 2 Materials and methods

### 2.1 Study design and patients

This retrospective case–control study enrolled consecutive patients aged ≥18 years who underwent overnight polysomnography (PSG) and MCG at the Shanghai Sixth People's Hospital Affiliated to Shanghai Jiao Tong University School of Medicine between June 2023 and October 2024. Based on predefined criteria, 78 patients with moderate-to-severe OSAS were assigned to the case group and 27 patients with normal-to-mild OSAS were assigned to the control group. Inclusion criteria comprised age ≥18 years, completion of ≥7-h PSG, and undergoing MCG. Exclusion criteria comprised significant comorbidities such as autoimmune diseases, malignancy, severe cardiac, hepatic or renal dysfunction, cardiomyopathies, macrovascular coronary disease, and conditions or medications affecting coronary microcirculation.

### 2.2 Diagnoses and definitions

Patients at high risk for OSAS based on typical symptoms, signs, and related comorbidities underwent nocturnal PSG (Alice, Netherlands) at the Department of Otorhinolaryngology of the Shanghai Sixth People's Hospital Affiliated to Shanghai Jiao Tong University School of Medicine to confirm the diagnosis of OSAS and evaluate disease severity. All recordings lasted at least 7 h. Data were collected from all participants, and diagnosis and severity stratification were performed in accordance with current guidelines using the apnea–hypopnea index (AHI) (14). An AHI of ≥5 to <15 events/h was classified as mild, ≥15 to <30 as moderate, and≥30 as severe OSAS. Hypertension was defined as systolic/diastolic blood pressure≥140/90 mmHg, current antihypertensive treatment, or prior diagnosis. Diabetes mellitus was diagnosed based on fasting blood glucose ≥7.0 mmol/L, 2-h oral glucose tolerance test (OGTT) ≥11.1 mmol/L, random blood glucose ≥11.1 mmol/L, or current use of glucose-lowering medications.

### 2.3 MCG examination

All the participants in this study were examined using a domestic unshielded nine-channel MCG device (MD-U041001, Mandi Medical). During the examination, the patients were in the supine position, and a superconducting quantum interference device (SQUID) was used to collect MCG signals to obtain MCG data and images. Participant MCG examination results were collected through the electronic medical record system. The obtained MCG data and images were processed and analyzed using the LabVIEW 2012 software.

### 2.4 Blood biochemical examination

Venous blood samples were collected from the patients in the case group and the control group. Laboratory tests were performed, including complete blood count, biochemical tests, blood lipid analysis, and myocardial injury markers. All tests were strictly conducted in accordance with the manufacturers’ instructions.

### 2.5 Statistical analysis

Categorical variables are presented as numbers and percentages (%). Between-group comparisons were performed using the Chi-square test or Fisher's exact test, as appropriate. Normality of continuous variables was assessed via the Shapiro–Wilk test. Normally distributed variables were described as mean ± standard deviation (SD), while non-normally distributed variables were presented as median [interquartile range: 25th percentile (P25)–75th percentile (P75)]. Between-group comparisons were conducted with the independent-samples t-test for normal data and the Mann–Whitney U test for non-normal data. MCG parameters that showed significant differences in univariate analysis were entered into multivariate logistic regression to calculate the odds ratio (OR) and corresponding 95% confidence interval (CI). We further constructed receiver operating characteristic (ROC) curves to evaluate the predictive performance of the candidate MCG parameters for moderate-to-severe OSAS patients. All statistical analyses were performed using SPSS version 26.0, and *p* < 0.05 was considered indicative of statistical significance.

## 3 Results

A total of 105 subjects were enrolled in this study based on the predefined inclusion and exclusion criteria. Among them, 78 patients with moderate-to-severe OSAS were assigned to the case group, and 27 patients with normal-to-mild OSAS were included in the control group. Since all participants included in this study underwent overnight PSG due to clinical OSAS symptoms or high clinical suspicion of OSAS, our study cohort had a relatively small sample size of patients with normal-to-mild OSAS. First, we analyzed traditional risk factors (baseline data and biochemical indicators) together with echocardiographic parameters of cardiac structure and diastolic function, which have been previously associated with microvascular impairment. Subsequently, we performed statistical analyses on MCG parameters to explore their relationship with OSAS severity. Finally, we screened and identified MCG indicators and a combined predictive model that could distinguish moderate-to-severe OSAS from normal-to-mild OSAS.

### 3.1 Comparison of baseline characteristics

The comparison results of the baseline data of the two groups of patients are detailed in Table 1. There were statistically significant differences in gender distribution, height, body weight, and body mass index (BMI) between the groups. In particular, the proportion of male patients in the case group was higher, and the height, body weight, and BMI were all significantly higher than those in the control group.

**Table 1:** Comparison of clinical features among different OSAS patient groups.

Variable	Normal-to-mild OSAS (*n* = 27)	Moderate-to-severe OSAS (*n* = 78)	*P*-value
Male, n (%)	14.00 (51.85)	67.00 (85.90)	**0** **.** **000**
Age (years)	32.00 (25.75–44.00)	36.50 (30.00–47.50)	0.119
Height (m)	169.04 ± 7.40	172.54 ± 7.87	**0**.**049**
Weight (kg)	67.15 ± 11.65	81.72 ± 14.61	**0**.**000**
BMI (kg/m^2^)	23.36 ± 3.63	27.32 ± 3.74	**0**.**000**
Smoking, n (%)	5 (18.52)	20 (25.64)	0.454
Drinking, n (%)	4 (14.81)	12 (15.38)	1.000
Diabetes, n (%)	0 (0.00)	2 (2.56)	0.550
Hypertension, n (%)	1 (3.70)	16 (20.51)	0.082

OSAS, obstructive sleep apnea syndrome.

### 3.2 Comparison of hematological, biochemical, and echocardiographic parameters

The overall statistical profiles of hematological, biochemical, and echocardiographic parameters for the two cohorts are summarized in Tables 2, 3. Statistical analysis revealed significant differences between the two groups in hemoglobin, glucose, alanine aminotransferase, creatinine, serum uric acid, triglyceride, high-density lipoprotein cholesterol (HDL-C), aspartate aminotransferase, lactate dehydrogenase, left atrial diameter (LA), mitral regurgitation (%), and E/A ratio >1 (%). Notably, all indicators except HDL-C concentration and E/A ratio > 1 (%) were significantly higher in the case group compared with the control group.

**Table 2:** Comparison of serum hematological and biochemical indexes among different OSAS patient groups.

Variable	Normal-to-mild OSAS (*n* = 27)	Moderate-to-severe OSAS (n = 78)	*p*-value
Hemoglobin (g/L)	140.43 ± 19.13	152.01 ± 14.89	**0** **.** **005**
Red blood cell (×10^12^/L)	4.58 (4.37–5.56)	5.13 (4.77–5.46)	0.059
Platelet (×10^9^/L)	252.50 (240.75–328.25)	256.00 (208.00–297.50)	0.360
White blood cell(×10^9^/L)	6.90 (4.98–8.00)	6.70 (5.85–7.95)	0.976
Kalium (mmol/L)	3.99 (3.86–4.22)	4.06 (3.93–4.34)	0.146
Natrium (mmol/L)	141.00 (139.75–143.50)	141.00 (139.00–142.00)	0.416
Chlorinum (mmol/L)	103.60 (101.48–105.05)	102.20 (100.70–104.00)	0.141
Glucose (mmol/L)	4.91 (4.58–5.15)	5.10 (4.83–5.65)	**0**.**001**
Glycated hemoglobin (%)	5.30 (5.10–5.43)	5.50 (5.20–5.90)	0.082
Alanine aminotransferase (U/L)	23.00 (17.75–33.25)	29.00 (25.00–55.50)	**0**.**000**
Aspartate aminotransferase (U/L)	28.00 (22.75–32.25)	29.00 (23.00–34.50)	**0**.**002**
Lactate dehydrogenase (U/L)	159.50 (142.00–169.00)	166.00 (150.00–203.00)	**0**.**027**
Creatinine (μmol/L)	66.30 (59.78–74.93)	74.70 (66.25–82.35)	**0**.**001**
Urea nitrogen (mmol/L)	4.68 ± 1.08	5.31 ± 1.20	0.244
Uric acid (μmol/L)	369.00 ± 134.99	413.07 ± 87.60	**0**.**012**
Triglyceride (mmol/L)	1.11 (0.89–1.50)	1.65 (1.22–2.16)	**0**.**000**
Total cholesterol (mmol/L)	4.86 ± 0.87	4.94 ± 0.96	0.773
High-Density Lipoprotein Cholesterol (mmol/L)	1.11 ± 0.22	1.04 ± 0.28	**0**.**015**
Low-density lipoprotein Cholesterol (mmol/L)	3.19 ± 0.76	3.18 ± 0.82	0.630

OSAS, obstructive sleep apnea syndrome.

**Table 3:** Comparison of cardiac ultrasound results between OSAS patient groups.

Variable	Normal-to-mild OSAS (*n* = 27)	Moderate-to-severe OSAS (*n* = 78)	*p*-value
LVEF (%)	66.00 (62.50–67.00)	64.00 (61.00–66.00)	0.136
LA (mm)	33.93 ± 4.23	35.10 ± 3.65	**0** **.** **006**
LVDD (mm)	46.00 (44.50–47.50)	47.00 (44.00–49.00)	0.091
E/A ratio >1, n (%)	25 (92.60)	42 (53.85)	**0**.**000**
Mitral regurgitation+, n (%)	12 (44.44)	51 (65.38)	**0**.**000**
Tricuspid regurgitation+, n (%)	19 (70.37)	55 (70.51)	0.989
Aortic valve+, n (%)	10 (37.04)	41 (52.56)	0.164
Abnormal ventricular wall motion+, n (%)	0 (0.00)	1 (1.28)	0.743

OSAS, obstructive sleep apnea syndrome; LVEF, left ventricular ejection fraction; LA, left atrial; LVDD, left ventricular end-diastolic diameter.

### 3.3 Comparison of MCG data

Significant intergroup differences in diastolic echocardiographic parameters (left atrial size, E/A ratio) confirmed subclinical diastolic dysfunction in the case group, providing a clinical basis for indirect identification of CMD in this population. Given that MCG has emerged as one of the core non-invasive modalities for CMD assessment, this study builds on preliminary findings to compare the distribution of characteristic MCG parameters among OSAS patients of varying disease severities. All MCG parameters were compared between the two groups, and detailed results are shown in Table 4. Thirty-five of the cardiac MCG parameters showed significant intergroup differences. In particular, qa, ttstpp, RS-mdama, and qr_rela_dist_neg_mode were greater in the control group, while the remaining parameters were significantly higher in the case group.

**Table 4:** Comparison of magnetocardiography among different groups of OSAS patients.

Variable	Normal-to-mild OSAS (*n* = 27)	Moderate-to-severe OSAS (*n* = 78)	*p*-value
dis-Q	4.804 ± 8.040	6.824 ± 10.918	0.380
dis-R	1.433 ± 3.512	1.800 ± 4.569	0.705
dis-S	5.567 ± 6.178	5.444 ± 7.247	0.937
dis-T	0.407 ± 1.474	1.212 ± 3.015	0.073
dis-QRS	10.789 ± 22.261	13.645 ± 25.634	0.608
dis-TT	1.137 ± 4.105	1.544 ± 4.102	0.658
QR-center1	0.160 ± 0.534	0.165 ± 0.707	0.975
QR-center2	0.244 ± 0.762	0.244 ± 0.955	0.997
QR-didma	64.237 ± 7.475	69.379 ± 8.289	**0** **.** **005**
QR-didav	56.344 ± 6.706	59.569 ± 5.516	**0**.**015**
QR-didmse	5.141 ± 2.073	5.631 ± 2.885	0.419
QR-dicd	64.115 ± 25.810	72.499 ± 31.948	0.221
QR-dicar	0.257 ± 0.162	0.314 ± 0.284	0.326
QR-wg	6.115 ± 2.486	5.885 ± 2.555	0.685
QR-pm	0.778 ± 4.042	3.631 ± 9.136	**0**.**030**
QR-nm	−62.896 ± 28.163	−66.477 ± 34.551	0.629
QR-r	42.785 ± 21.012	41.265 ± 23.742	0.769
QR-v	2.211 ± 1.028	2.017 ± 1.072	0.414
QR-mse	1.310 ± 0.611	1.319 ± 0.586	0.947
QR-v1	2.199 ± 1.183	2.005 ± 1.198	0.467
QR-mse1	1.128 ± 0.651	1.093 ± 0.554	0.788
qa	293.681 ± 34.031	260.953 ± 92.878	**0**.**010**
ra	49.033 ± 15.209	74.200 ± 72.954	**0**.**005**
qra	120.304 ± 36.393	125.071 ± 45.411	0.623
QR-areaf	0.204 ± 0.110	0.236 ± 0.167	0.271
QR-aread	0.549 ± 0.169	0.613 ± 0.251	0.222
QR-epma	12.985 ± 5.464	14.909 ± 6.272	0.159
QR-epav	9.305 ± 1.157	10.016 ± 1.199	**0**.**009**
QR-epmse	1.311 ± 0.947	1.664 ± 1.335	0.208
QR-enma	22.990 ± 7.282	21.541 ± 7.420	0.382
QR-enav	10.311 ± 0.973	10.231 ± 1.256	0.762
QR-enmse	3.604 ± 1.643	3.365 ± 1.564	0.502
RS-center1	0.017 ± 0.394	−0.046 ± 0.563	0.597
RS-center2	0.045 ± 0.553	−0.038 ± 0.763	0.606
RS-didma	69.467 ± 11.855	76.964 ± 11.449	**0**.**004**
RS-didav	59.096 ± 7.674	64.087 ± 6.934	**0**.**002**
RS-didmse	4.944 ± 2.082	6.460 ± 3.181	**0**.**006**
RS-dicd	74.137 ± 24.854	81.537 ± 33.663	0.298
RS-dicar	0.347 ± 0.217	0.410 ± 0.301	0.324
RS-wg	6.133 ± 3.050	6.287 ± 2.841	0.812
RS-pm	24.193 ± 44.433	28.447 ± 37.698	0.631
RS-nm	−74.144 ± 48.656	−63.804 ± 46.824	0.330
RS-r	38.333 ± 19.846	38.718 ± 23.624	0.940
RS-v	1.973 ± 0.971	1.910 ± 0.938	0.765
RS-mse	1.444 ± 0.809	1.403 ± 0.722	0.805
RS-v1	2.036 ± 1.447	1.947 ± 1.093	0.739
RS-mse1	1.066 ± 0.645	1.109 ± 0.731	0.787
sa	198.015 ± 79.437	228.717 ± 51.674	0.069
rsa	139.030 ± 56.586	143.941 ± 49.232	0.668
RS-areaf	0.205 ± 0.144	0.195 ± 0.137	0.747
RS-aread	0.560 ± 0.232	0.788 ± 0.373	**0**.**000**
RS-epma	17.660 ± 9.696	20.340 ± 9.308	0.205
RS-epav	10.213 ± 1.253	10.695 ± 1.101	0.061
RS-epmse	2.037 ± 1.855	2.676 ± 2.061	0.158
RS-enma	21.170 ± 10.740	18.895 ± 9.758	0.311
RS-enav	10.081 ± 1.356	10.084 ± 1.824	0.993
RS-enmse	3.163 ± 2.122	2.563 ± 1.836	0.163
TT-center1	0.129 ± 0.642	0.352 ± 0.642	0.121
TT-center2	0.180 ± 0.905	0.501 ± 0.897	0.114
TT-didma	66.585 ± 7.701	71.892 ± 10.585	**0**.**019**
TT-didav	62.030 ± 5.903	65.541 ± 8.047	**0**.**040**
TT-didmse	2.385 ± 1.623	3.395 ± 2.548	**0**.**020**
TT-dicd	20.904 ± 16.112	36.700 ± 35.166	**0**.**027**
TT-dicar	0.023 ± 0.049	0.038 ± 0.053	0.202
TT-wg	1.867 ± 0.756	2.464 ± 2.081	0.149
TT-pm	4.981 ± 13.233	5.238 ± 10.799	0.920
TT-nm	−1.026 ± 4.169	−3.031 ± 8.772	0.120
TT-r	15.378 ± 8.507	21.854 ± 13.995	**0**.**006**
TT-v	0.252 ± 0.125	0.334 ± 0.173	**0**.**024**
TT-mse	0.459 ± 0.098	0.569 ± 0.272	**0**.**003**
TT-v1	0.255 ± 0.138	0.330 ± 0.170	**0**.**041**
TT-mse1	0.455 ± 0.093	0.534 ± 0.242	**0**.**018**
ta	56.730 ± 14.221	69.313 ± 56.119	0.253
tt50	0.022 ± 0.116	0.090 ± 0.201	**0**.**037**
tt75	0.378 ± 0.386	0.569 ± 0.289	**0**.**024**
tta	18.241 ± 13.691	24.623 ± 15.275	0.058
TT-areaf	0.099 ± 0.064	0.145 ± 0.091	**0**.**006**
TT-aread	0.131 ± 0.125	0.193 ± 0.137	**0**.**044**
TT-epma	11.779 ± 2.310	12.578 ± 4.714	0.401
TT-epav	10.053 ± 1.316	10.546 ± 1.824	0.200
TT-epmse	0.696 ± 0.688	0.926 ± 1.209	0.353
TT-enma	11.597 ± 2.404	12.158 ± 2.864	0.363
TT-enav	10.149 ± 0.873	10.258 ± 1.429	0.641
TT-enmse	0.604 ± 0.514	0.837 ± 0.744	0.134
QR-qca	211.974 ± 37.146	205.300 ± 64.000	0.514
QR-qia	86.189 ± 25.765	86.032 ± 22.849	0.976
QR-rca	301.359 ± 59.007	291.573 ± 68.861	0.511
QR-ria	94.341 ± 21.875	96.473 ± 15.304	0.580
QR-iama	132.578 ± 30.280	132.263 ± 25.612	0.958
QR-iaav	91.685 ± 10.364	91.479 ± 8.383	0.918
QR-iamse	17.515 ± 7.585	18.891 ± 7.785	0.427
QR-qra	118.041 ± 53.901	119.241 ± 54.501	0.921
QR-mctd	53.448 ± 32.405	60.572 ± 36.509	0.371
QR-mcar	0.215 ± 0.186	0.237 ± 0.238	0.665
QR-areac	0.212 ± 0.102	0.240 ± 0.158	0.284
QR-dpma	66.059 ± 33.079	70.405 ± 35.259	0.576
QR-dpav	28.881 ± 12.765	30.117 ± 13.034	0.671
QR-dpmse	16.215 ± 9.477	16.510 ± 9.350	0.888
QR-dvma	42.370 ± 19.534	43.137 ± 18.324	0.854
QR-dvav	19.856 ± 11.549	17.835 ± 7.538	0.302
QR-dvmse	11.056 ± 4.685	11.582 ± 5.440	0.655
QR-mdama	100.715 ± 32.410	109.791 ± 41.608	0.306
QR-mdaav	52.007 ± 24.292	49.817 ± 21.296	0.658
QR-mdamse	28.459 ± 10.751	31.012 ± 14.312	0.399
RS-sca	160.733 ± 66.636	154.046 ± 56.791	0.615
RS-sia	88.622 ± 24.171	87.117 ± 17.753	0.731
RS-iama	132.230 ± 26.875	131.226 ± 28.756	0.874
RS-iaav	91.685 ± 8.204	91.760 ± 8.994	0.970
RS-iamse	17.874 ± 8.115	17.833 ± 8.077	0.982
RS-rsa	148.293 ± 60.634	146.953 ± 54.628	0.915
RS-mctd	67.396 ± 30.773	77.951 ± 36.053	0.177
RS-mcar	0.371 ± 0.384	0.433 ± 0.370	0.460
RS-areac	0.155 ± 0.086	0.199 ± 0.153	0.068
RS-dpma	80.578 ± 33.825	92.487 ± 46.037	0.221
RS-dpav	25.741 ± 9.568	29.942 ± 12.634	0.118
RS-dpmse	16.744 ± 7.887	20.454 ± 11.477	0.068
RS-dvma	65.930 ± 27.300	54.427 ± 26.158	0.054
RS-dvav	17.648 ± 8.919	15.600 ± 7.117	0.231
RS-dvmse	14.659 ± 6.643	12.312 ± 6.230	0.100
RS-mdama	145.667 ± 40.535	126.101 ± 44.903	**0**.**048**
RS-mdaav	46.804 ± 21.173	42.812 ± 17.311	0.333
RS-mdamse	37.459 ± 12.768	32.464 ± 14.599	0.117
TT-iama	100.511 ± 22.197	101.132 ± 22.862	0.903
TT-iaav	84.756 ± 8.040	82.236 ± 13.650	0.368
TT-iamse	6.678 ± 5.808	8.921 ± 6.770	0.128
TT-tta	22.378 ± 19.908	34.586 ± 33.688	**0**.**027**
TT-mctd	25.748 ± 20.773	54.700 ± 70.064	**0**.**002**
TT-mcar	0.067 ± 0.160	0.093 ± 0.222	0.573
TT-areac	0.108 ± 0.073	0.174 ± 0.131	**0**.**002**
TT-dpma	39.433 ± 35.663	46.253 ± 40.661	0.441
TT-dpav	20.078 ± 9.019	23.069 ± 14.651	0.217
TT-dpmse	6.756 ± 7.276	8.486 ± 9.598	0.395
TT-dvma	18.885 ± 11.377	24.778 ± 23.084	0.207
TT-dvav	9.389 ± 4.627	11.149 ± 9.625	0.364
TT-dvmse	4.063 ± 3.307	5.231 ± 6.254	0.357
TT-mdama	54.007 ± 36.510	62.673 ± 45.593	0.325
TT-mdaav	27.270 ± 17.970	29.613 ± 23.312	0.636
TT-mdamse	12.093 ± 13.051	12.977 ± 11.534	0.741
qsitv	88.300 ± 13.358	89.680 ± 12.374	0.625
htrt	72.036 ± 9.566	78.387 ± 11.449	**0**.**011**
tpnrt1	1.352 ± 0.363	1.297 ± 0.445	0.565
tpnrt2	1.325 ± 0.338	1.250 ± 0.377	0.367
rtrt	3.958 ± 1.697	5.181 ± 4.464	0.169
rtrt3	7.001 ± 7.968	9.959 ± 17.151	0.390
tnirt	5.453 ± 4.273	7.670 ± 5.512	0.060
tnirt1	1.017 ± 1.416	2.320 ± 5.217	0.204
qrsttrt	14.623 ± 16.213	27.964 ± 53.619	0.207
stsl	18.024 ± 10.283	14.359 ± 8.369	0.068
stsl1	0.118 ± 0.117	0.074 ± 0.073	0.076
ttflt	1.528 ± 0.129	1.590 ± 0.177	0.095
ttflt1	1.641 ± 0.063	1.635 ± 0.109	0.761
ttstpp	4.908 ± 4.470	2.917 ± 2.668	**0**.**036**
ttstpn	9.432 ± 10.145	5.242 ± 6.807	0.018
strs	0.778 ± 0.429	0.700 ± 0.358	0.359
tsm	2.466 ± 1.321	5.323 ± 18.546	0.427
tsm1	0.646 ± 0.389	1.372 ± 1.408	**0**.**000**
tmdf	32.070 ± 14.207	49.220 ± 25.262	**0**.**000**
tmdf1	37.110 ± 20.616	54.630 ± 27.637	**0**.**003**
stfs	5.325 ± 1.288	5.764 ± 2.379	0.364
qrs_abs_angle_pos_mode	38.284 ± 24.555	41.438 ± 24.494	0.566
qrs_abs_magni_neg_mode	0.185 ± 0.280	0.223 ± 0.293	0.551
qrs_abs_magni_pos_mode	0.435 ± 0.314	0.425 ± 0.346	0.897
qrs_angle_minmax_min	−88.646 ± 49.608	−93.577 ± 54.315	0.679
qrs_angle_minmax_mode	−40.149 ± 68.774	−41.030 ± 68.465	0.954
qrs_ch_ratio_a4_mean	0.327 ± 0.024	0.318 ± 0.033	0.133
qrs_max_abs_magni_mode	0.397 ± 0.261	0.383 ± 0.286	0.821
qrs_max_rela_disp_median	0.097 ± 0.030	0.095 ± 0.030	0.799
qrs_max_rela_disp_mode	0.000 ± 0.000	0.015 ± 0.036	0.030
qrs_max_x_position_max	7.111 ± 0.866	7.356 ± 0.935	0.234
qrs_max_x_position_median	5.930 ± 0.802	6.200 ± 0.898	0.169
qrs_max_x_position_mode	5.941 ± 1.106	6.097 ± 1.277	0.571
qrs_max_y_position_max	6.504 ± 1.180	6.881 ± 1.458	0.228
qrs_max_y_position_min	3.374 ± 0.805	3.368 ± 0.951	0.976
qrs_max_y_position_mode	4.470 ± 0.766	4.529 ± 1.004	0.781
qrs_rela_dist_neg_mode	5.437 ± 2.109	4.679 ± 2.714	0.191
qrs_rpeak_angle_minmax	−49.060 ± 14.342	−40.864 ± 43.431	0.149
qrs_rpeak_y_min_mfm	1.896 ± 1.299	2.226 ± 1.909	0.408
qrs_x_max_mfm_max	6.522 ± 1.151	7.314 ± 1.617	**0**.**008**
qrs_x_max_mfm_mode	4.307 ± 1.024	4.418 ± 1.942	0.779
qrs_y_max_mfm_mode	5.185 ± 2.436	5.556 ± 3.345	0.598
qrs_y_min_mfm_mode	2.441 ± 2.575	3.027 ± 3.108	0.381
qr_abs_angle_neg_median	21.992 ± 15.048	24.919 ± 15.302	0.392
qr_abs_angle_neg_min	7.786 ± 9.391	8.831 ± 11.146	0.664
qr_abs_angle_pos_min	35.500 ± 16.835	30.648 ± 9.219	0.247
qr_abs_magni_neg_mode	0.039 ± 0.115	0.166 ± 0.296	**0**.**002**
qr_abs_magni_pos_mode	0.385 ± 0.357	0.319 ± 0.325	0.378
qr_angle_minmax_median	−22.348 ± 37.376	−20.000 ± 36.333	0.774
qr_ch_ratio_a4_mean	0.467 ± 0.066	0.450 ± 0.075	0.284
qr_max_angle_min	−5.489 ± 87.893	−22.942 ± 93.208	0.397
qr_max_angle_mode	−5.489 ± 87.893	−22.942 ± 93.208	0.397
qr_max_angle_range	139.033 ± 97.879	150.286 ± 104.818	0.626
qr_max_rela_disp_max	2.112 ± 1.598	2.235 ± 1.562	0.726
qr_max_rela_disp_median	0.108 ± 0.048	0.102 ± 0.041	0.561
qr_max_rela_disp_mode	0.024 ± 0.046	0.035 ± 0.050	0.293
qr_max_rela_disp_range	2.112 ± 1.598	2.233 ± 1.561	0.731
qr_max_rela_disp_skewness	2.678 ± 1.576	2.803 ± 1.554	0.719
qr_max_rela_disp_std	0.459 ± 0.340	0.487 ± 0.334	0.710
qr_max_x_position_median	5.839 ± 0.691	6.146 ± 0.919	0.115
qr_max_x_position_mode	5.933 ± 0.896	6.186 ± 1.102	0.286
qr_max_y_position_min	3.900 ± 0.826	3.909 ± 0.954	0.965
qr_max_y_position_mode	4.378 ± 0.757	4.572 ± 1.079	0.390
qr_qpeak_max_angle	74.420 ± 114.817	64.246 ± 112.813	0.688
qr_qpeak_x_max_mfm	4.519 ± 1.624	4.815 ± 2.003	0.489
qr_rela_dist_neg_mode	5.489 ± 2.376	4.235 ± 2.962	**0**.**031**
qr_x_min_mfm_median	7.891 ± 0.892	8.166 ± 1.356	0.236
qr_y_min_mfm_median	3.598 ± 2.639	4.187 ± 2.715	0.331
qr_y_min_mfm_min	1.178 ± 1.439	1.349 ± 1.747	0.649
qr_y_min_mfm_mode	2.463 ± 2.803	3.442 ± 3.514	0.150
qr_y_min_mfm_range	6.363 ± 1.561	6.135 ± 2.107	0.607
rs_abs_angle_neg_mode	30.579 ± 28.435	24.163 ± 26.092	0.284
rs_abs_angle_pos_min	24.613 ± 17.463	18.463 ± 14.895	0.080
rs_abs_angle_pos_mode	39.642 ± 24.790	37.678 ± 25.085	0.726
rs_abs_magni_neg_mode	0.255 ± 0.353	0.257 ± 0.295	0.977
rs_abs_magni_pos_mode	0.260 ± 0.317	0.346 ± 0.339	0.252
rs_angle_minmax_max	91.484 ± 74.871	100.727 ± 66.612	0.549
rs_angle_minmax_min	−85.826 ± 51.269	−80.122 ± 69.079	0.695
rs_angle_minmax_mode	−29.615 ± 77.904	−39.494 ± 88.066	0.606
rs_ch_ratio_a4_mean	0.481 ± 0.052	0.457 ± 0.071	0.068
rs_max_abs_magni_mode	0.386 ± 0.277	0.392 ± 0.348	0.929
rs_max_angle_max	99.687 ± 47.958	89.784 ± 74.205	0.430
rs_max_angle_range	184.568 ± 90.943	197.446 ± 96.086	0.544
rs_max_rela_disp_median	0.089 ± 0.049	0.092 ± 0.045	0.718
rs_max_rela_disp_mode	0.004 ± 0.019	0.014 ± 0.035	0.059
rs_max_x_position_mode	5.974 ± 1.195	5.812 ± 1.511	0.573
rs_max_y_position_max	6.085 ± 1.201	6.327 ± 1.555	0.464
rs_max_y_position_min	3.656 ± 0.864	3.606 ± 0.852	0.797
rs_y_max_mfm_median	5.889 ± 1.624	5.955 ± 2.264	0.890
rs_y_max_mfm_min	2.830 ± 2.255	2.009 ± 1.882	0.067
rs_y_max_mfm_mode	5.248 ± 2.411	5.396 ± 3.529	0.810
rs_y_min_mfm_length	24.740 ± 3.948	25.170 ± 7.558	0.711
rs_y_min_mfm_max	6.256 ± 2.012	6.800 ± 1.858	0.202
rs_y_min_mfm_mean	3.777 ± 1.261	4.019 ± 1.433	0.438
rs_y_min_mfm_median	3.437 ± 1.606	3.448 ± 1.798	0.978
rs_y_min_mfm_min	1.552 ± 1.235	1.967 ± 1.797	0.269
rs_y_min_mfm_mode	3.889 ± 2.581	3.238 ± 2.754	0.285
t_tpeak_y_min_mfm	1.615 ± 0.975	1.858 ± 1.749	0.495
t_tt_abs_angle_neg_max	17.611 ± 7.097	19.527 ± 11.866	0.321
t_tt_abs_angle_neg_median	13.065 ± 7.883	14.963 ± 11.247	0.420
t_tt_abs_angle_neg_min	10.516 ± 7.972	11.127 ± 10.339	0.780
t_tt_rela_dist_neg_skewness	3.202 ± 2.245	2.926 ± 2.472	0.610
t_tt_rela_dist_pos_skewness	2.728 ± 1.805	2.881 ± 2.462	0.732
t_tt_y_min_mfm_max	2.304 ± 1.152	2.412 ± 1.746	0.766
t_tt_y_min_mfm_mean	1.618 ± 0.914	1.902 ± 1.725	0.418
t_tt_y_min_mfm_median	1.598 ± 0.961	1.890 ± 1.749	0.412
t_tt_y_min_mfm_min	1.233 ± 0.934	1.424 ± 1.594	0.558
t_tt_y_min_mfm_mode	1.456 ± 1.047	1.837 ± 1.846	0.311

OSAS, obstructive sleep apnea syndrome.

### 3.4 Identification of MCG parameters predicting moderate-to-severe OSAS via logistic analysis

All MCG parameters with significant between-group differences in univariate analysis were entered into multivariate logistic regression to identify independent predictors for moderate-to-severe OSAS. Four parameters were confirmed as independent risk factors: RS-didma (OR = 1.071, *p* = 0.011, 95% CI: 1.016–1.128), htrt (OR = 1.060, *p* = 0.025, 95% CI: 1.007–1.079), tmdf (OR = 1.015, *p* = 0.004, 95% CI: 1.015–1.079), and qr_rela_dist_neg_mode (OR = 0.762, *p* = 0.012, 95% CI: 0.616–0.943). Detailed results are shown in Table 5.

**Table 5:** Multivariate logistic regression analysis of moderate-to-severe OSAS.

Variable	*Β* value	Wald *χ*^2^	OR	95% CI	*p*-value
RS-didma	0.068	6.478	1.071	1.016–1.128	**0.011**
htrt	0.058	5.026	1.060	1.007–1.079	**0**.**025**
tmdf	0.045	8.275	1.015	1.015–1.079	**0**.**004**
qr_rela_dist_neg_mode	−0.272	6.277	0.762	0.616–0.943	**0**.**012**

OSAS, obstructive sleep apnea syndrome.

### 3.5 ROC analysis for evaluating predictive performance of MCG parameters in moderate-to-severe OSAS patients

ROC curve analysis was performed to evaluate the predictive efficacy of MCG parameters in patients with moderate-to-severe OSAS. The area under the receiver operating characteristic curve (AUC) values for RS-didma, htrt, tmdf, and qr_rela_dist_neg_mode were 0.675 (95% CI: 0.552–0.797, *p* = 0.007), 0.677 (95% CI: 0.563–0.791, *p* = 0.006), 0.712 (95% CI: 0.608–0.817, *p* = 0.001), and 0.627 (95% CI: 0.512–0.742, *p* = 0.0499), respectively; the corresponding optimal cutoff values were 66.35, 75.7575, 43.000, and 4.300. Furthermore, the combined model incorporating three MCG parameters (htrt, tmdf, and RS-didma) yielded an AUC of 0.797 (95% CI: 0.705–0.889, p < 0.001) and outperformed any single MCG parameter in predictive performance. The resulting ROC curve is presented in Figure 1, and detailed information including AUC values, optimal cutoff thresholds, sensitivity, and specificity are summarized in Table 6.

**Figure 1:**
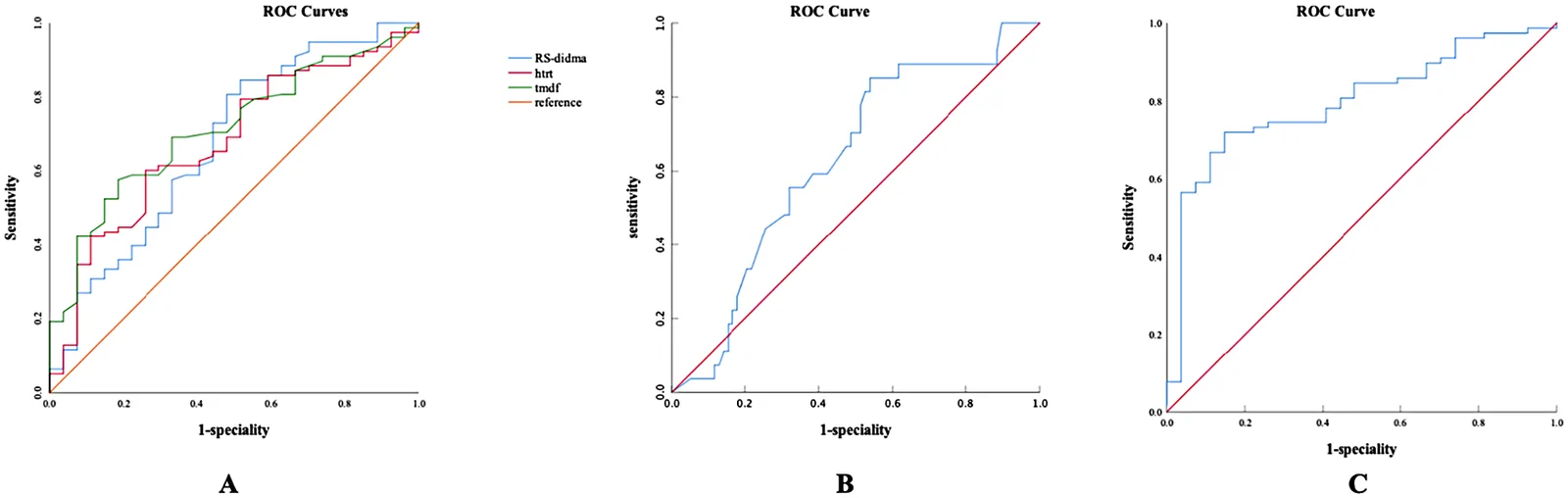
ROC curves of MCG parameters for predicting moderate-to-severe OSAS: **(A)** ROC curves of RS-didma, htrt, and tmdf; **(B)** ROC curve of qr_rela_dist_neg_mode; and **(C)** combined ROC curve of the RS-didma, htrt, and tmdf MCG parameters. AUC, area under the curve; CI, confidence interval.

**Table 6:** ROC analysis of statistically significant MCG parameters for predicting the severity of OSAS.

Variable	AUC	95% CI	Sensitivity	Specialty	Cutoff	*p*-value
RS-didma	0.675	0.552–0.797	0.808	0.519	66.35	**0** **.** **007**
htrt	0.677	0.563–0.791	0.603	0.741	75.7575	**0**.**006**
tmdf	0.712	0.608–0.817	0.577	0.815	43.0000	**0**.**001**
qr_rela_dist_neg_mode	0.627	0.512–0.742	0.852	0.462	4.3000	**0**.**0499**
combined	0.797	0.705–0.889	0.718	0.852	0.718226	**0**.**000**

OSAS, obstructive sleep apnea syndrome; MCG, magnetocardiography.

## 4 Discussion

This study aimed to evaluate the value of MCG parameters in distinguishing moderate-to-severe OSAS from normal-to-mild OSAS in PSG-confirmed patients. Our analyses identified four MCG parameters—RS-didma, htrt, tmdf, and qr_rela_dist_neg_mode—that were significantly associated with moderate-to-severe OSAS in this cohort. ROC curve analysis confirmed that each of the four parameters had modest discriminative value for distinguishing OSAS severity, and we further constructed a combined prediction model integrating three parameters (htrt, tmdf, and RS-didma). The combined model achieved an AUC of 0.797 for differentiating moderate-to-severe from normal-to-mild OSAS, with a sensitivity of 0.808 and a specificity of 0.519. Overall, our study suggests that this panel of MCG-derived parameters may aid in risk stratification among OSAS patients, particularly those with moderate-to-severe disease, though further validation is needed before routine clinical application.

In this study, we observed that the male gender, higher BMI, hemoglobin, fasting blood glucose, creatinine, uric acid, and triglyceride levels were significantly associated with increased severity of OSAS, consistent with previous studies (15–19). The association with male sex is thought to reflect androgenic effects on respiratory control and a greater propensity for upper-airway fat deposition (17). Obesity, the most well-established risk factor, reduces pharyngeal airway caliber and stability while increasing chest wall load and reducing lung compliance, thereby exacerbating nocturnal breathing disturbances (20). Elevated hemoglobin raises blood viscosity, worsens tissue hypoxia and vascular damage, and sets up a vicious cycle that intensifies with increasing OSAS severity (21). Fasting glucose, uric acid, and triglycerides—core components of the metabolic syndrome—exhibit a bidirectional relationship with OSAS; they may aggravate OSAS severity through systemic endothelial dysfunction, enhanced inflammation, and impaired airway neuromuscular regulation (16). The link between elevated creatinine and OSAS severity is likely mediated by chronic renal vasoconstriction, endothelial injury, and interstitial hypoxia induced by long-standing OSAS, culminating in mild renal impairment and reduced creatinine clearance (22). The consistency of these traditional risk associations with prior work supports the reliability of our cohort and provides a solid basis for the subsequent analyses. In this study, we observed that the moderate-to-severe OSAS group had a higher proportion of E/A ratio <1 and more pronounced left atrial enlargement compared to the normal-to-mild group. These findings suggest that early echocardiographic changes in diastolic function are already prevalent in patients with more severe OSAS.

OSAS is an independent risk factor for CAD progression, with IH serving as the core pathological driver of CMD. Recurrent IH promotes oxidative stress and excessive reactive oxygen species production, which impairs endothelial function and reduces nitric oxide bioavailability. In addition, IH activates matrix metalloproteinases, triggering concentric arteriolar remodeling, and the marked swings in intrathoracic pressure induce abnormal LV wall stress, promoting vascular smooth muscle hypertrophy and microvascular structural changes that culminate in CMD (23). The association between OSAS severity and CMD risk is further supported by evidence that coronary flow reserve (CFR) declines in a graded fashion with increasing AHI (9); indeed, a meta-analysis confirmed that patients with severe OSAS have significantly lower CFR than those without OSAS. Moreover, a cohort study using positron emission tomography (PET)-derived myocardial flow reserve (MFR) found that the risk of MFR <1.5 was more than double in severe OSAS compared with non-severe disease (24). Such chronic microcirculatory compromise can induce myocardial ischemia, accelerate atherosclerosis, and increase the risks of heart failure and sudden death. Our echocardiographic analysis revealed statistically significant intergroup differences in LA size and the E/A ratio, two established markers of cardiac diastolic function, indicating subclinical early diastolic dysfunction in participants with moderate-to-severe OSAS. Consistent with prior findings that diastolic dysfunction indirectly reflects CMD, our results confirmed that CMD develops at an early disease stage in this population. These echocardiographic findings provided a robust rationale for our subsequent investigation of myocardial magnetic signal characteristics using MCG, and laid the foundation for identifying specific MCG-based biomarkers of CMD in patients with OSAS.

MCG utilizes SQUID technology to non-invasively detect cardiac magnetic fields at the picotesla level, overcoming biophysical limitations of conventional electrocardiography (25). By capturing eddy currents and tangential currents, MCG provides unique functional assessment for CAD and myocardial ischemia (25). In CAD diagnosis, a study of 291 stable CAD patients established an MCG scoring model with significant efficacy (AUC = 0.824), demonstrating superior specificity (87.9%) compared with CT-derived fractional flow reserve (CT-FFR) (26). The MAGNETO trial further validated its clinical utility, confirming reliable diagnostic performance for suspected myocardial ischemia within a 90-s protocol (27). However, before this study, no studies had examined MCG features in OSAS populations across different risk strata. To address this unmet research gap, the present study characterized the MCG signatures of the OSAS population; this not only provides a simple diagnostic modality for early, rapid, and accurate clinical diagnosis of patients with moderate-to-severe OSAS but also offers reference data for the subsequent identification of CMD related to moderate-to-severe OSAS via MCG.

Our analysis confirmed that each of the four individual MCG parameters had measurable discriminative value for moderate-to-severe OSAS, and the combined model (RS-didma, htrt, tmdf) achieved the best performance (AUC = 0.797). These findings support the auxiliary risk stratification value of MCG in this setting. The QRS-related parameters, RS-didma and qr_rela_dist_neg_mode, primarily reflect alterations in QRS amplitude or spatial characteristics. As the QRS complex represents ventricular depolarization, changes in its morphology, duration, or voltage often point to myocardial abnormalities such as ischemia, fibrosis, or conduction disturbances. Thus, these parameters may indicate ventricular electrical remodeling in patients with moderate-to-severe OSAS, consistent with the early diastolic function changes seen on echocardiography. One possible mechanism could involve chronic intermittent hypoxia-induced myocardial structural and electrophysiological alterations that affect depolarization synchrony and conduction pathways, although this remains speculative. The T-wave-derived parameters (htrt and tmdf) are associated with ventricular repolarization. Abnormalities in T-wave morphology, direction, or amplitude reflect increased repolarization dispersion and electrical instability. Alterations in these indices suggest global repolarization abnormalities and correlate with OSAS severity. A possible explanation involves chronic ischemia-induced metabolic changes in cardiomyocytes, affecting ion channel function and leading to altered action-potential duration and increased repolarization heterogeneity (25); however, this mechanism requires confirmation in future studies. Although the precise electrophysiological pathways linking these MCG parameters to OSAS severity and diastolic function changes require further investigation, our findings, as components of a non-invasive predictive model, offer a valuable basis for early identification of high-risk OSAS patients and thus may help guide timely intervention.

To the best of our knowledge, this is the first study to investigate the association between MCG parameters and moderate-to-severe OSAS, and their correlations with echocardiographic diastolic function indicators. Several limitations should be considered. First, the single-center retrospective design limits generalizability, and prospective multicenter validation is needed. Second, despite the acceptable overall performance of our combined model, the modest sample size resulted in wide 95% confidence intervals for the odds ratios of the four independent predictors, reducing estimation precision; sample size imbalance between groups may also have affected regression stability and limited the individual predictive power of single parameters. Third, the echocardiographic parameters (E/A ratio and LA size) used in this study are descriptive observations of diastolic function in OSAS patients; they should be interpreted with caution and do not represent diagnostic measures for any specific underlying pathology. Fourth, the model's specificity was low (0.519), corresponding to a false-positive rate exceeding 40%, which curtails its utility as a standalone diagnostic tool. Accordingly, this model is more appropriate for screening than confirmatory diagnosis and would benefit from combination with other clinical or laboratory variables to improve diagnostic accuracy in practice. Finally, the precise electrophysiological mechanisms underlying the observed MCG alterations in patients with moderate-to-severe OSAS remain incompletely understood, and further mechanistic studies with larger, adequately powered cohorts are warranted to validate and extend our findings.

## 5 Conclusions

This study demonstrates that specific MCG parameters are independently associated with moderate-to-severe OSAS. These non-invasive MCG markers show promising performance for early risk stratification in this high-risk population, but their real-world clinical utility requires rigorous validation in large prospective cohorts. The distinct MCG phenotypic alterations observed in our cohort indicate that the MCG can capture disease severity-related electrophysiological changes, although the underlying mechanisms remain to be elucidated. Future large-scale prospective studies are warranted to identify the specific MCG signatures of CMD in patients with moderate-to-severe OSAS.

## Data Availability

The raw data supporting the conclusions of this article will be made available by the authors without undue reservation.
